# Effect of DON and ZEN and their metabolites DOM-1 and HZEN on B cell proliferation and antibody production

**DOI:** 10.3389/fimmu.2024.1338937

**Published:** 2024-02-21

**Authors:** Alix Pierron, Alexandra Kleber, Elisabeth Mayer, Wilhelm Gerner

**Affiliations:** ^1^ Department of Pathobiology, Institute of Immunology, University of Veterinary Medicine, Vienna, Austria; ^2^ dsm-firmenich, Animal Nutrition and Health R&D Center, Tulln, Austria

**Keywords:** deoxynivalenol, de-epoxy-deoxynivalenol, zearalenone, hydrolyzed zearalenone, B cells, immune system

## Abstract

**Introduction:**

The mycotoxins deoxynivalenol (DON) and zearalenone (ZEN), produced by *Fusarium* fungi, are frequently found in the cereal-rich diet of pigs and can modulate the immune system. Some enzymes or bacteria present in the digestive tract can de-epoxydize DON to deepoxy-deoxynivalenol (DOM-1) and biotransform ZEN into hydrolyzed ZEN (HZEN). The effects of these metabolites on immune cells, particularly with respect to the vaccine responses, are poorly documented. The aim of this study was to address the impact of DON and ZEN and their respective derivatives, on proliferation, and antibody production of porcine B cells *in vitro*.

**Methods:**

Peripheral blood mononuclear cells (PBMCs), isolated from healthy pigs, were stimulated with the Toll-like receptor (TLR) 7/8-agonist Resiquimod (R848) or the TLR/1/2-agonist Pam3Cys-SKKKK in combination with DON [0.1-1.6 µM] or DOM-1 [1.6 µM and 16 µM] and ZEN [2.5-40 µM] or HZEN [40 µM].

**Results:**

A strong decrease in B-cell proliferation was observed at DON concentrations equal to or exceeding 0.8 µM and at ZEN concentrations equal to or exceeding 20 µM. Treatment with 1.6 µM DON or 40 µM ZEN led to almost a complete loss of live CD79α^+^ B cells. Moreover, CD21 expression of proliferating IgG^+^ and IgM^+^ B-cell subsets was decreased at DON concentrations equal to and exceeding 0.4 µM and at ZEN concentrations equal to or exceeding 10 µM. ELISpot assays revealed a decrease of IgG-secreting B cells at concentrations of and exceeding 0.4 µM and at ZEN concentrations equal to and exceeding 10 µM. ELISA assays showed a decrease of IgM, IgG, and IgA secretion at concentrations equal to or exceeding 0.4 µM DON. ZEN reduced IgM secretion at 20-40 µM (both R848 and Pam3Cys-SKKKK), IgG secretion at 40 µM (both R848 and Pam3Cys-SKKKK) and IgA secretion at 20-40 µM.

**Discussion:**

Our *in vitro* experiments show that while DON and ZEN impair immunoglobulin production and B-cell proliferation, this effect is abrogated by HZEN and DOM-1.

## 1 Introduction

As the most frequently occurring natural contaminants of food and feed, mycotoxins pose significant health threats to both humans and animals. A worldwide survey has confirmed mycotoxin contamination in more than 70% of agricultural commodities (1). Particularly prominent among the array of fungal metabolites are the fusariotoxins deoxynivalenol (DON) and zearalenone (ZEN) (2, 3), which do not only occur separately, but have recently been reported to frequently co-occur in feedstuffs and in complete feed for pigs (4, 5).

Depending on the severity and duration of exposure, the trichothecene DON can inflict acute (e.g. diarrhea, emesis, leucocytosis, haemorrhage, endotoxemia) as well as chronic (growth retardation, immunosuppression) toxicological effects (6). Its interaction with the peptidyl transferase center of the 60S ribosomal subunit induces a “ribotoxic stress” response, leading to the inhibition of elongation during protein synthesis. This causes the activation of mitogen-activated protein kinases (MAPKs), resulting in oxidative stress, local inflammation, and modulation of the immune response (6–8). ZEN on the other hand, is primarily known for its toxicity on the reproductive system, which is based on its structural similarity to estrogen, enabling it to induce activation of estrogen receptors α (ERα) and β (ERβ) (9). While *in vitro* studies confirm ZEN-induced proliferation of estrogen-dependent cells (e.g. MCF-7) (10), *in vivo* investigations report hyperestrogenism and severe reproductive disorders (e.g. compromised fertility, abnormal fetal development, swelling/reddening of the vulva, metaplasia of the uterus) following exposure to the mycotoxin (2, 11, 12). However, toxicology of ZEN goes far beyond the latter effects (2, 11, 13, 14), ranging from histopathological alterations with subsequent development of liver cancer (15, 16), to hematotoxic impacts (disruption of coagulation, modification of blood parameters) (15, 17, 18), to genotoxic effects (*in vitro* formation of DNA adducts, DNA fragmentation, micronucleus formation, chromosomal aberration, cell proliferation, and cell apoptosis) (15, 18).

Although the toxicological profiles of DON and ZEN are characterized in various studies, their effects on immune cells, particularly with respect to vaccine-related immune responses, has not been investigated in detail. Available *in vitro* investigations with porcine lymphocytes and other immune-related cells, confirm that DON impairs critical functions of these cells, including their survival, proliferation, and maturation (19–22). A comparative study of DON and DOM-1 confirmed DON-induced impairment of proliferation in concanavalin A (ConA) stimulated bovine, porcine, and chicken peripheral blood mononuclear cells (PBMCs), as well as a strong DON-induced reduction of CD4^+^, CD8^+^, and γδ T cell proliferation (19). Similarly, flow cytometry phenotyping has revealed DON-induced reduction of proliferation of major porcine T-cell subsets (CD4^+^, CD8^+^, and γδ T cells) as well as a reduction of the expression of co-stimulatory molecules CD27 and CD28 (21), which are critical for T-cell activation, proliferation, and survival (23, 24). DON has also been reported to modulate the expression of transcription factors and related cytokines, suggesting that for CD4^+^ and CD8^+^ T cells in particular, DON can modulate T cell differentiation into a pro-inflammatory type-1 direction, which could be favorable or unfavorable for ongoing immune responses to infection or vaccination (25). Furthermore, Toutounchi et al., 2021 investigated the effects of DON exposure on the development of allergies and vaccine responsiveness in a mouse model. It was shown that exposure to DON during pregnancy and lactation can lead to an imbalanced state of the immune system, resulting in increased allergic immune responses to food allergens and a decreased immune response to vaccination against influenza virus (26).

Like DON, also ZEN has significant effects on immune responses with immunostimulatory or immunosuppressive results (27). Despite the increasing number of studies analyzing ZEN-induced immune modulation, data are fragmentary, particularly with respect to vaccine-related effects. Most available studies confirm a ZEN-mediated reduction of serum IgG and IgM, regardless of the animal species (mice, rats, swine), toxin concentration, or duration of exposure (28–36). It is postulated that ZEN exposure may interfere with the ability to uphold an adequate immune response to vaccination and that the toxin can alter antibody synthesis to vaccine antigens. While some studies report decrease of antibody titer to porcine parvovirus (31) or swine plague vaccination (35) in ZEN-treated animals, more research is required to better understand the effects of ZEN on the underlying vaccine-related immune responses.

Feed additives leading to the biodegradation of DON and ZEN are currently available to reduce bioavailability and/or toxicity of these mycotoxins. DON is biotransformed to DOM-1 via the cleavage of the 12,13-epoxy ring by an epoxidase of the Gen. nov. (formerly Eubacterium) sp. nov. BBSH 797 of the Coriobacteriaceae family, isolated from bovine rumen fluid (37). With respect to ZEN, the bacterial enzyme zearalenone hydrolase (ZenA), applied as a feed additive, degrades ZEN in the gastrointestinal tract (38, 39), releasing hydrolyzed zearalenone (HZEN), which partially decarboxylates spontaneously to decarboxylated hydrolyzed ZEN (DHZEN). For the safe implementation of DOM-1 and HZEN, the continuous assessment of both metabolites is necessary. Concerning DOM-1, a few studies have confirmed its detoxified status compared to DON (19, 40–43). However, with respect to HZEN, only one study has shown a significantly reduced estrogenic activity of HZEN *in vitro* and in female pigs (44). No studies are yet available focusing on the effects of HZEN on the immune system.

Thus, this study presents the first investigation of the effects of the mycotoxins DON and ZEN as well as their derivatives DOM-1 and HZEN on proliferation and antibody production of porcine B cells. Antibodies, produced by terminally differentiated B cells (plasma cells and plasmablasts), are the most frequent correlate of protection in vaccines (45). Hence, we focused on *in vitro* experiments investigating the proliferation of B cells and major subsets therein as well as antibody production by ELISA and ELISpot. Particularly with respect to ZEN and HZEN our study delivers much needed information. It does not only provide valuable data regarding the detrimental effects of DON and ZEN on major functional B-cell parameters but also underlines the safety of the biodegradation products DOM-1 and HZEN, both of which did not affect the investigated immune parameters.

## 2 Material and methods

### 2.1 Mycotoxins and stimulants

Mycotoxins and their metabolites were supplied by Biopure (Romer Labs^®^, Tulln, Austria) and had a purity of ≥ 99%. Deoxynivalenol (DON) and deepoxy-deoxynivalenol (DOM-1) were dissolved in sterile water to obtain a 5 mM stock solution. Zearalenone (ZEN) and hydrolyzed-zearalenone (HZEN) were dissolved in sterile water and DMSO (1:1 dilution ratio) to obtain a 5 mM stock solution (46). Stock solutions were stored in aliquots at -20°C.

For B cell stimulation, PBMC cultures were treated either with the Toll-like receptor (TLR) 7/8-agonist Resiquimod (InvivoGen, Toulouse, France) or the TLR 1/2-agonist Pam3Cys-SKKKK (EMC microcollections GmbH, Tübingen, Germany) in the concentrations outlined below.

### 2.2 Isolation of peripheral blood mononuclear cells

For the isolation of PBMCs, blood of six 6-months-old pigs was obtained from an abattoir, which slaughters animals from different conventional finishing farms. Animals were subjected to electric high voltage anesthesia followed by exsanguination. This procedure is in accordance with the Austrian Animal Welfare Slaughter Regulation. Blood was collected during exsanguination into heparinized sample tubes. PBMCs were isolated by gradient centrifugation (Pancoll human, density: 1.077 g/mL, PAN Biotech, Aidenbach, Germany) and frozen at -150°C for further use.

### 2.3 Proliferation assays by violet proliferation staining

Prior to *in vitro* treatment, PBMCs were labelled with the CellTrace™ Violet Cell Proliferation Kit (Thermo Fisher Scientific, Waltham, MA), as described by Reutner et al. (2012) (47). Subsequently, labelled PBMCs [2 × 10^5^/well] were cultivated in cell culture medium (RPMI1640 with stable glutamine [PAN-Biotech], supplemented with 10% [v/v] heat-inactivated fetal calf serum [FCS, Gibco™, Thermo Fisher Scientific]), with or without Pam3Cys-SKKKK [10 µM] or R848 [2.5 µg/ml] stimulation and in the presence or absence of DON [0.1-1.6 µM], DOM-1 [1.6 µM], ZEN [2.5-40 µM], or HZEN [40 µM] for 4 days at 37°C. Per condition, at least four wells were prepared (quadruplicates). Subsequently, wells treated with the same conditions were pooled and centrifuged (350g, 10 minutes room temperature). Supernatants were collected and stored at -20°C until use in ELISA. Cell pellets were resuspended and subjected to B-cell phenotyping and proliferation analysis by flow cytometry (FCM) as outlined below.

### 2.4 Phenotyping of B cells by FCM

Cells derived from *in vitro* cultures were labelled with primary antibodies (IgM and IgG) and second step reagents, as listed in **Table 1**. Cells were stained by incubation with the respective antibodies and second step reagents for 20 minutes at room temperature. Between incubation steps, cells were washed twice with PBS (PAN-Biotech) supplemented with 3% FCS. Prior to the third incubation, cells were washed only with PBS, to allow staining with a live/dead discrimination dye (Fixable Viability dye eFluor780, Thermo Fisher Scientific). In the same step free binding sites of secondary antibodies were blocked with mouse IgG (Jackson Immuno Research, Ely, UK, 1μg per sample). Following this, cells were again washed twice with PBS + 3% FCS and stained for CD21. Prior to the last incubation cells were washed again, subsequently fixed and permeabilized using the BD Cytofix/CytopermTM Fixation/Permeabilization Kit (BD Biosciences, San Jose, CA) according to manufacturer’s instructions. Fixed and permeabilized cells were then stained for intracellular CD79α expression for 30 minutes at 4°C. FCM analyses of stained cells were performed on a FACS Canto II (BD Biosciences, San Jose, CA) flow cytometer equipped with three lasers (405, 488, and 633 nm). Per sample, at least 100,000 lymphocytes were collected, based on FSC/SSC properties. Data analyses of flow cytometric raw data were performed by FACS Diva 6.1.3 and FlowJo version 10.4 (both BD Biosciences). Percentages of live PBMCs were identified by gating on all cellular events, as shown in **Supplementary Figure 1**). The gating strategies for proliferating B cells and B-cell subsets are also illustrated in **Supplementary Figure 1**.

**Table 1:** Antibodies used for B-cell staining in FCM.

Antigen	Clone	Isotype	Fluorochrome	Labeling strategy	Primary Ab source
CD79α	HM57	IgG1	APC	Directly conjugated	Dako
CD21	B-ly4	IgG1	Brilliant Violet 605	Directly conjugated	BD Biosciences
IgG	MT424	IgG2a	PE-Cy7	Biotin–streptavidin^a^	Mabtech
IgM	K52 1C3	IgG1	Alexa488	Secondary ab^b^	Bio-Rad

a Streptavidin-PE-Cy7, Thermo Fisher Scientific.

b Goat anti-mouse IgG1-Alexa488, Thermo Fisher Scientific.

### 2.5 Quantification of antibody secretion by ELISA

Total concentrations of major Ig classes (IgM, IgG, and IgA) in the culture supernatants of treated PBMCs were determined via ELISA (Bethyl, Interchim, Montluçon, France), which was performed according to manufacturer’s instructions and as previously described (48). Optical densities were measured in a Sunrise ELISA reader (Tecan, Crailsheim, Germany) at 450 nm. Standard curves were used to convert the optical densities to cytokine concentrations in the supernatants.

### 2.6 IgG B-cell ELISpot

ELISpot analysis (ELISpot Flex Porcine IgG [ALP], Mabtech) was performed according to the manufacturer’s instructions and as previously described (49). Briefly, three replicates of 2 × 10^5^ cells/well were stimulated with PAM-3Cys-SKKKK or R848 and treated with DON [0.1-1.6 µM], DOM-1 [1.6 µM, 16 µM], ZEN [2.5-40 µM], or HZEN [40 µM] in cell culture medium (as above) for three days. Following treatment, cells were harvested, washed three times with PBS (PAN-Biotech) and resuspended in cell culture medium. Cells were then counted and transferred to B-cell ELISpot plates [2.5 × 10^4^ cells/well] for an incubation period of 4 hours at 37°C. Subsequently, plates were washed with PBS and subjected to the detection antibody MT424 (Mabtech) for 2 hours at room temperature. Streptavidin-ALP (dilution: 1:2000, Roche, Vienna, Austria) was added for 1 hour at room temperature, followed by a 5-bromo-4-chloro-3-indolyl phosphate/nitro blue tetrazolium substrate, which was left to incubate in the dark for 5 minutes. Spots were counted with an AID ELISpot reader (AID, Strassberg, Germany).

### 2.7 Statistical analysis

Graphs were created and statistic calculations performed with Graphpad Prism V7.04 (GraphPad Software, San Diego, CA). The data were subjected to a one-way ANOVA followed by a Bonferroni’s multiple comparison test, with a p value < 0.05 considered as significant. In case data was not normally distributed, the Kruskall-Wallis test was applied.

## 3 Results

### 3.1 DON and ZEN affect survival of activated PBMCs

Following R848 or Pam3Cys-SKKKK stimulation of PBMCs in the presence or absence of DON [0.1-1.6 µM], DOM-1 [1.6 µM, 16 µM], ZEN [2.5-40 µM], or HZEN [40 µM] for four days, cells were harvested, labelled for B-cell phenotyping and subsequently analyzed by flow cytometry. Based on light scatter properties total intact cells were gated, followed by live cell analysis (**Supplementary Figure 2**).

**Figure 1:**
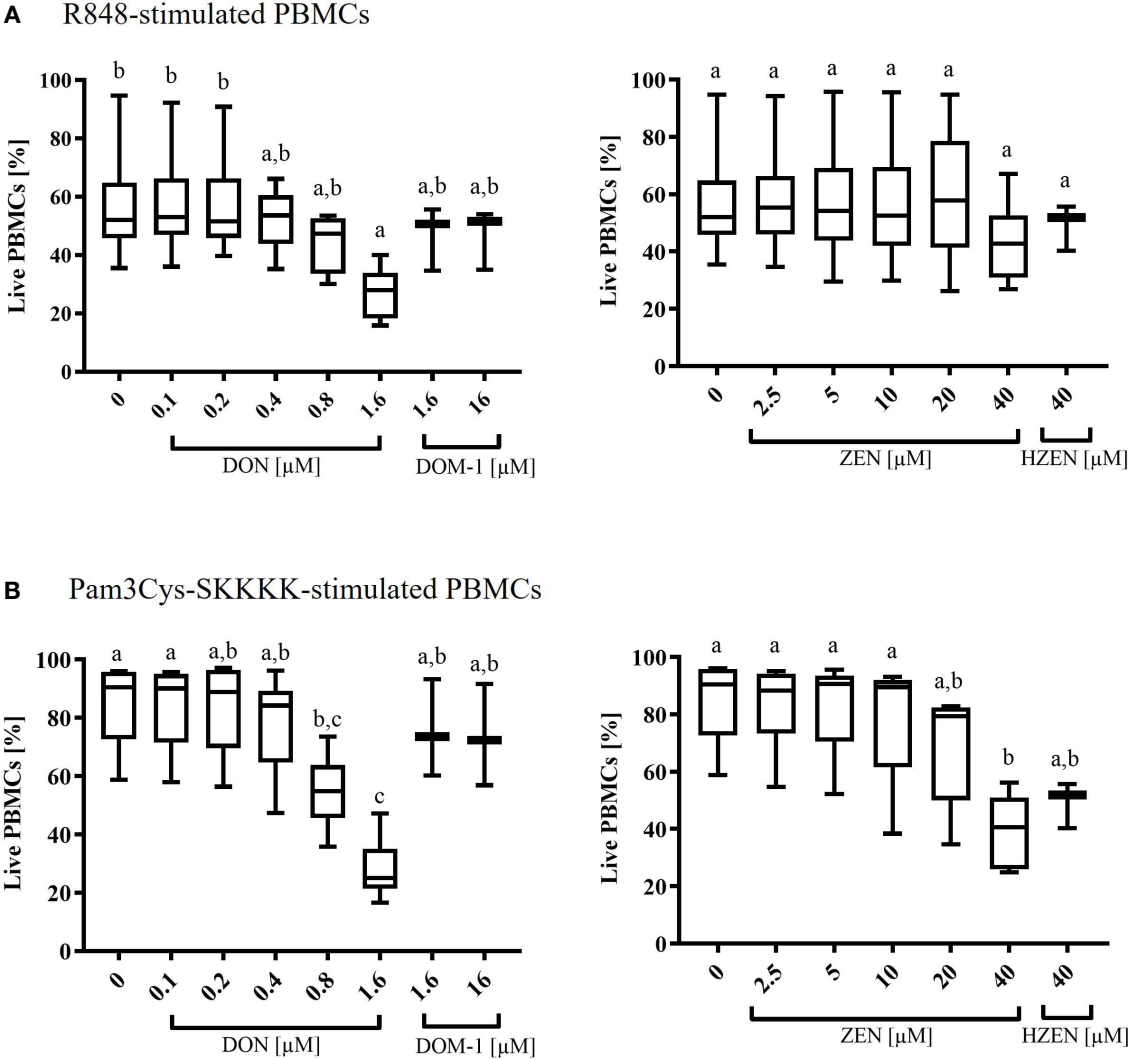
Percentages of live **(A)** R848- or **(B)** Pam3Cys-SKKKK-stimulated PBMCs in the presence of deoxynivalenol (DON) and zearalenone (ZEN) and their metabolites deepoxy-deoxynivalenol (DOM-1) and hydrolyzed zearalenone (HZEN). PBMCs stained with a violet proliferation dye were exposed to either of two stimuli (R848 or Pam3Cys-SKKKK) in the absence or presence of DON, DOM-1, ZEN or HZEN for a period of 4 days. Cells were then harvested and analyzed by flow cytometry. Boxplots display the percentage of live R848 or Pam3Cys-SKKKK stimulated PBMCs in presence of different mycotoxins concentrations. Different letters indicate significant differences between treatments (One Way ANOVA, Bonferroni *post-hoc* test, p<0.05, n=6 for the mycotoxins and n=3 for the metabolites).

Under R848-stimulation, the survival rate of PBMCs (percentage of live cells) was significantly decreased at 1.6 µM DON (p=0.0319) only (**Figure 1A**). Under Pam3Cys-SKKKK stimulation, the cell survival rate was significantly reduced when cells were subjected to 0.8 µM (p=0.0341) and 1.6 µM (p<0.0001) DON (**Figure 1A**) or 40 µM ZEN (p=0.001) (**Figure 1B**). Neither DOM-1 [1.6-16 µM] nor HZEN [40 µM] had any effect on the survival rate of PBMCs, regardless of the stimulus used (**Figures 1A, B**).

The baseline response to DON, DOM-1, ZEN, and HZEN in the absence of a TLR-agonist was also investigated and results are shown in **Supplementary Figure 3**. Overall, survival rates in non-stimulated cultures were high but significantly reduced in the presence of DON (0.8 µM, 1.6 µM) and ZEN (20 µM, 40 µM). Again, DOM-1 and HZEN had no influence on cell survival.

### 3.2 DON and ZEN affect proliferation of total B cells and B-cell subsets

To evaluate total B-cell proliferation by FCM, dead cells were excluded as shown in **Supplementary Figures 1, 2**. This was followed by gating for CD79α^+^ total B cells and subsequently CD79α^+^ B cells were analyzed for violet proliferation dye fluorescence (**Supplementary Figures 1, 4**) to identify total proliferating B cells. Proliferation dye fluorescence profiles were also combined with expression profiles of CD21, IgM or IgG to identify proliferating CD21^+^, IgM^+^ or IgG^+^ within total B cells, respectively (**Supplementary Figures 1, 5**).

**Figure 2:**
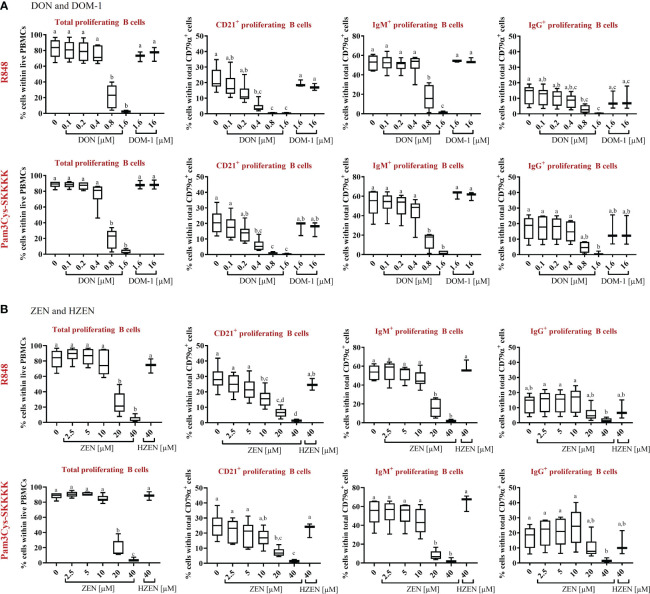
Percentages of total proliferating B cells and major subsets (CD21^+^, IgM^+^, and IgG^+^) in the presence or absence of **(A)** DON [0.1-1.6 µM] and DOM-1 [1.6 and 16 µM] or **(B)** ZEN [2.5-40 µM] and HZEN [40 µM] under R848 or Pam3Cys-SKKKK stimulation. Violet proliferation dye-stained PBMCs were stimulated with R848 or Pam3Cys-SKKKK and cultivated in the absence or presence of DON, DOM-1, ZEN, or HZEN for four days. After harvesting, cells were labelled for CD79α, CD21, IgM, and IgG. Gates were applied to identify proliferating total B cells or B-cell subsets within total B cells. Different letters indicate significant differences between treatments (One Way ANOVA, Bonferroni *post-hoc* test, p<0.05, n=6 for the mycotoxins and n=3 for the metabolites).

Compared to the control, total B-cell proliferation was significantly reduced in the presence of DON [0.8-1.6 µM] (p<0.0001), regardless of the applied stimulus (**Figure 2A**). Furthermore, DON-induced proliferation reductions were observed in all R848-stimulated B-cell subsets. DON led to reductions of CD21^+^ proliferating cells at concentrations of 0.4-1.6 µM (p<0.0001), IgM^+^ proliferating B cells at 0.8-1.6 µM (p<0.0001), and IgG^+^ proliferating B cells at 0.8 µM (p=0.0347) and 1.6 µM (p=0.002). Similarly, DON led to significant proliferation reductions in all Pam3Cys-SKKK stimulated B-cell subsets. DON significantly reduced CD21^+^ proliferating cells at 0.4 µM (p=0.0004), 0.8 µM, and 1.6 µM (both: p<0.0001) as well as IgM^+^ proliferating cells at 0.8 and 1.6 µM (both: p<0.0001). Regarding IgG^+^ proliferating cells, a DON induced reduction was observed at 0.8 (p=0.0565) and 1.6 µM (p=0.0029), however significance levels were only reached for the latter concentration. DOM-1 [1.6 and 16 µM] had no reducing effect on total B-cell proliferation or on the proliferation of the different B-cell subsets, regardless of the applied stimulus (**Figure 2A**).

With respect to ZEN, total B-cell proliferation was significantly reduced at a concentration of and exceeding 20 µM (p<0.0001), regardless of the stimulus applied (**Figure 2B**). ZEN-induced proliferation reductions were detected in all R848-stimulated B-cell subsets. ZEN significantly reduced CD21^+^ proliferating B cells at 10 µM (p=0.0069), 20 µM (p<0.0001) and 40 µM (p<0.0001) and IgM^+^ proliferating B cells at 20 µM and 40 µM (both: p<0.0001). With respect to IgG^+^ proliferating B cells, ZEN-induced reductions were observed at 40 µM (p= 0.0586), however significance levels were not reached. Similar effects of ZEN were observed in Pam3Cys-SKKKK-stimulated B cells. ZEN led to significant reductions of CD21^+^ proliferating cells at 20 µM (p=0.0004) and 40 µM (p<0.0001), IgM^+^ proliferating cells at 20 and 40 µM (both p<0.0001) and IgG^+^ proliferating cells at 40 µM (p=0.0446). HZEN (40 µM) had no reducing effect on total B-cell proliferation or on the proliferation of different B-cell subsets, regardless of the applied stimulus (**Figure 2B**).

The effect of DON, DOM-1, ZEN and HZEN in the absence of TLR-agonists R848 and Pam3CysSKKKK stimulated cells was also investigated (**Supplementary Figure 6**). The observed effects were similar to those seen in stimulated cells, but on a much lower level since spontaneous proliferation of B cells and B-cell subsets is low. Due to this, significant differences in reduction of B-cell proliferation seen with DON and ZEN in R848 or Pam3Cys-SKKKK stimulated cultures were not always reproducible in non-stimulated cultures.

### 3.3 DON and ZEN affect the number of IgG-secreting cells

**Figure 3:**
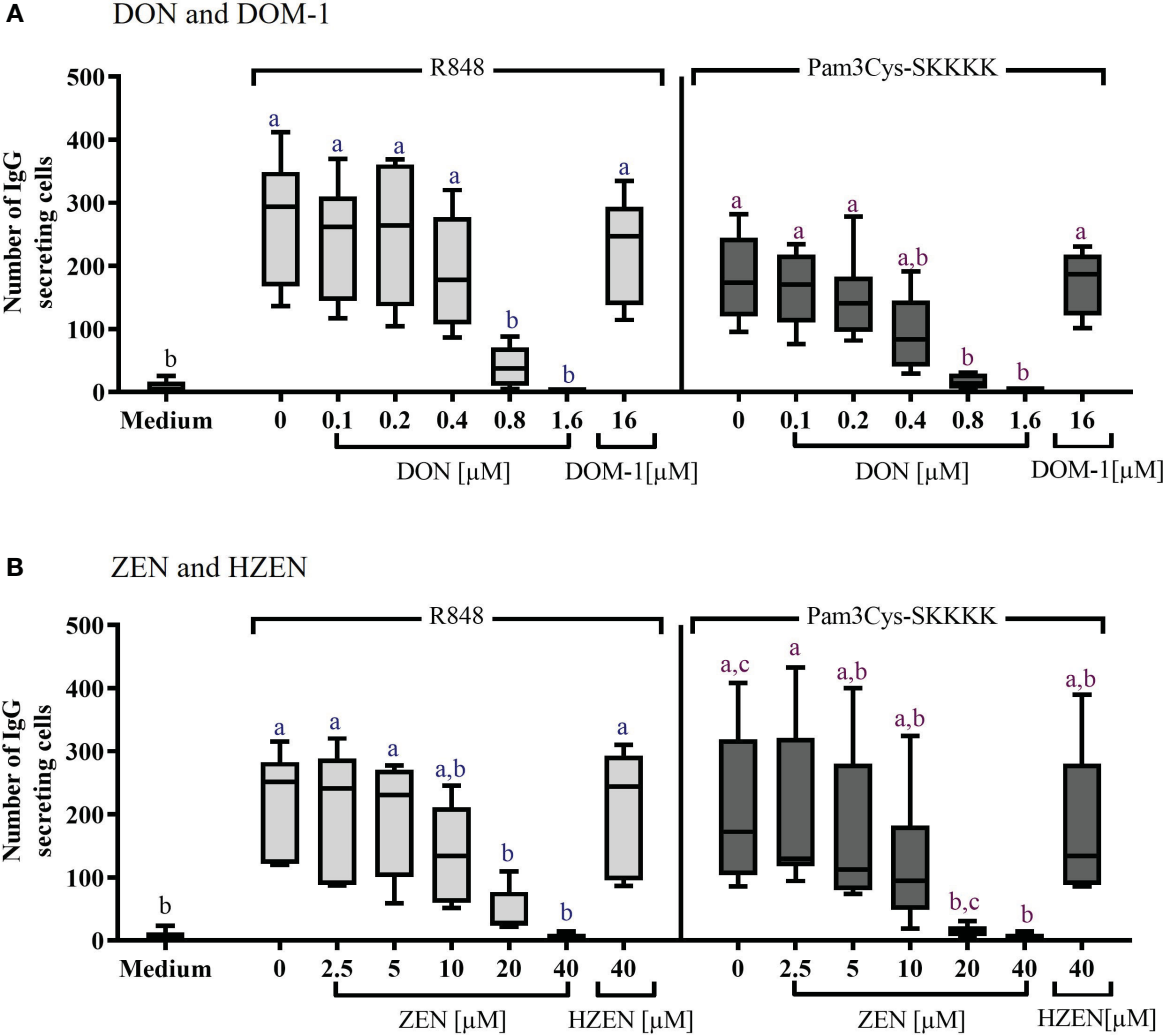
Number of IgG-secreting cells after stimulation with R848 or Pam3Cys-SKKKK and treatment with **(A)** DON [0.1-1.6 µM], DOM-1 [16 µM], **(B)** ZEN [2.5-40 µM], or HZEN [40 µM]. PBMCs were stimulated with R848 or Pam3Cys-SKKKK and cultivated in the absence or presence of DON, DOM-1, ZEN, or HZEN for three days. Thereafter, cells were transferred into ELISpot plates and IgG-secreting cells were quantified. Different letters indicate significant differences between the medium and treatments for each stimulation (One Way ANOVA, Bonferroni *post-hoc* test, p<0.05, n=6).

The effects of DON, DOM-1, ZEN, and HZEN on the number of IgG secreting B cells was assessed via ELISpot assays (**Figure 3**). Spots in such assays indicate that IgG was released and captured by anti-IgG antibodies at this particular spot, indicating the presence of an IgG secreting cell (**Supplementary Figure 7**). Stimulation of PBMCs with R848 or Pam3Cys-SKKKK led to a significant increase of IgG-secreting cells. DON [0.8-1.6 µM] (**Figure 3A**) and ZEN [20-40 µM] (**Figure 3B**) induced a significant decrease of IgG-secreting cells, regardless of the stimulus applied. Neither DOM-1 nor HZEN had any effect on the of IgG secreting cells.

### 3.4 DON and ZEN reduce concentrations of IgM, IgG, and IgA in cell culture supernatants

**Figure 4:**
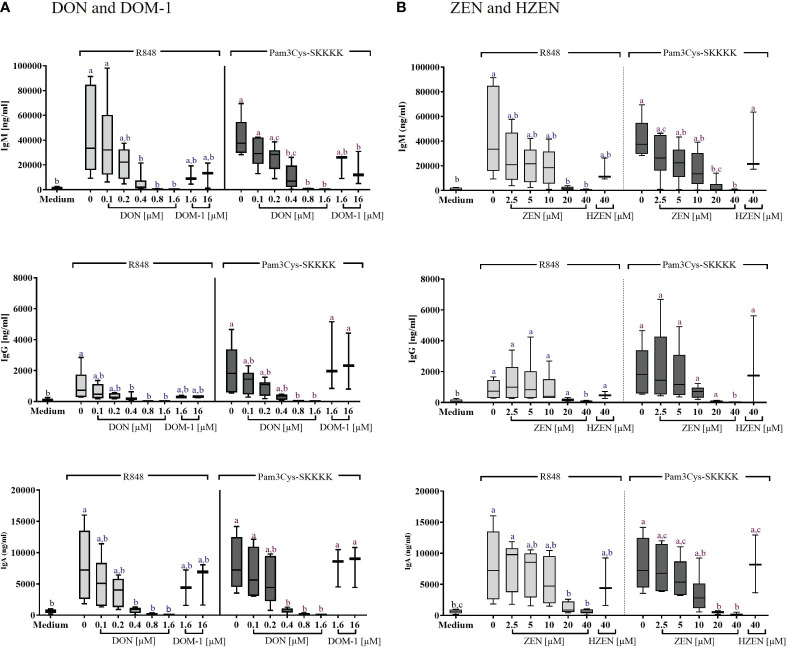
Concentrations of IgM, IgG, and IgA antibodies in the supernatant of R848 or Pam3CysSKKK stimulated PBMCs, following treatment with **(A)** DON [0.1-1.6 µM] or DOM-1 [1.6 and 16 µM] as well as **(B)** ZEN [2.5-40 µM] or HZEN [40 µM]. PBMCs were stimulated for 4 days with R848 or Pam3Cys-SKKKK in the presence or absence of the metabolites DON, DOM-1, ZEN, or HZEN. Antibody concentrations were determined in cell culture supernatants by ELISA. Different letters indicate significant differences between the medium control and treatments within each stimulation (One Way ANOVA, Bonferroni *post-hoc* test, p<0.05, n=6 for the mycotoxins and n=3 for the metabolites).

The concentrations of IgM, IgG, and IgA in the supernatants of stimulated B cells, cultivated for FCM, were determined by ELISA. Significant increases of IgM, IgG, and IgA secretion were detected in supernatants of cells stimulated with either R848 or Pam3Cys-SKKKK, compared to cells exposed to cell culture medium alone (**Figures 4A, B**).

DON led to a significant reduction in IgM, IgG, and IgA levels of R848 stimulated B cells between 0.4 and 1.6 µM. In Pam3Cys-SKKKK stimulated B cells, IgM and IgA levels were reduced significantly between 0.4 and 1.6 µM DON, while IgG levels of Pam3Cys-SKKKK stimulated B cells were reduced significantly between 0.8 and 1.6 µM DON. DOM-1 [1.6 and 16 µM] did not induce significant changes of antibody concentrations in any of the applied treatments (**Figure 4A**).

ZEN significantly reduced IgM levels in supernatants of cells stimulated with R848 at a concentration of 20 µM (p=0.0027) and 40 µM (p=0.0018). Under Pam3Cys-SKKKK stimulation, ZEN induced a significant IgM reduction at 20 µM and 40 µM (both p<0.0001). Furthermore, IgG levels were decreased significantly by 40 µM ZEN (R848: p=0.0184; Pam3Cys-SKKKK: p=0.0019), regardless of the stimulus applied. Finally, ZEN significantly reduced IgA levels at both 20 µM (R848: p=0.0371; Pam3Cys-SKKKK: p=0.0009) and 40 µM (R848: p=0.0147; Pam3Cys-SKKKK: 0.0005), regardless of the stimulus used. Treatment with HZEN [40 µM] did not induce significant changes of antibody concentrations in any of the applied treatments (**Figure 4B**).

Effects of DON, DOM-1, ZEN and HZEN on the levels of IgM, IgG, and IgA in the supernatants of non-stimulated cells **Supplementary Figure 8**) showed similar results as in R848- and Pam3CysSKKK stimulated cells, however at a lower level, with significant reductions only seen for some conditions with high DON concentrations.

## 4 Discussion

The potential detrimental effects of the *Fusarium*-derived mycotoxins DON and ZEN have been extensively explored and documented, from both *in vitro* and *in vivo* perspectives (6, 12, 44, 50). Due to the consumption of cereal-rich feed, pigs – in particular – are heavily affected by both of these mycotoxins (12, 51). While acute DON consumption can cause diarrhea, emesis, leucocytosis, hemorrhage, endotoxemia and, ultimately, shock-like death, chronic low-dose exposure results in growth retardation and immunological impairments (6, 51). ZEN on the other hand, is predominantly known for inducing hyperestrogenism, causing diverse clinical symptoms including, among others, swelling and reddening of the vulva, metaplasia of uterus, ovarian atrophy, enlargement of the mammae, and reduced fertility (2, 11, 12).

The concentrations of DON and ZEN used throughout this study closely reflect the physiologically relevant concentrations *in vivo*. DON is absorbed in the upper gastrointestinal tract up to 100% suggesting a nearly complete systemic absorption and can be identified in the serum, exposing immune cells (52). Furthermore, Döll et al, 2003 (53) and Dänicke et al., 2004 (54) reported a comparable and linear relationship, with concentrations of up to 100 ng/mL DON (~ 0.3 µM) and 325 ng/mL DON (~1 µM) detected in serum. The systemic bioavailability of ZEN was reviewed to vary between 78 and 87% suggesting that just 22 to 13% of an oral bolus are excreted via feces and the rest gets in contact with immune cells (55). Studies have reported maximal plasma concentrations up to 15 µg/mL (47 µM) ZEN after oral ingestion of ZEN via feed (56). The metabolites DOM-1 and HZEN were tested in equivalent, but physiological not relevant concentrations, to example the non-toxic effects thereof. Overall, several studies have shown that especially DON and ZEN contamination in animal feed, even though regulated, are very common. In 74,821 samples of feed and feed raw materials (e.g., maize, wheat, soybean) collected from 100 countries, 88% of the samples were contaminated with at least one mycotoxin. In feed, maximal concentrations of DON was 84,860 µg/kg and of ZEN 105,000 µg/kg - making them both the most relevant and ubiquitously found mycotoxins (3).

Despite the substantial amount of information which has been generated for DON and ZEN-related health risks, detailed knowledge regarding impacts on immune parameters, particularly in the context of vaccine-related immune responses, is still lacking. We therefore investigated the influence of DON and ZEN on the proliferation and antibody production of porcine B cells, with antibody production ultimately resulting in humoral immunity. Our investigations uncovered detrimental effects of DON and ZEN on the survival rate of stimulated PBMCs as well as proliferation of total B cells and selected B-cell subsets (CD21^+^, IgM^+^, IgG^+^). In line with these findings, our results show that both DON and ZEN significantly decrease the number of IgG-secreting B cells as well as the secretion of IgM, IgG and IgA when measured in the supernatants of stimulated B cells. Finally, all analyses in our study include a direct comparison between the effects of DON and its metabolite DOM-1 as well as the impacts of ZEN and its detoxification product HZEN. For all tested parameters we report the absence of impairing effects of both metabolites, DOM-1 and HEZN on diverse parameters of B-cell functionality.

In order to analyze the effects of DON and ZEN, as well as their metabolites, PBMCs were stimulated using two commercially available Toll-like receptor (TLR) agonists, R848 (Resiquimod) and Pam3CysSKKKK. While R848 is a low molecular weight synthetic molecule which activates immune cells via the TLR7/TLR8 MyD88-dependent signaling pathway (57, 58), Pam3Cys-SKKKK is a synthetic analogue of naturally occurring lipoproteins, known for its activation of TLR 1/2. For both molecules it has been shown that they are powerful stimulators of porcine B cells, driving activation (CD25 up-regulation), proliferation and Ig-production (59). Effects of DON, DOM-1, ZEN, and HZEN on cell survival, proliferation of total B cells and B cell subsets, as well as production of Ig classes were also investigated in unstimulated cells (i.e. without TLR-agonist) (**Supplementary Figures 3, 6, 8**). It was clearly shown that the overall results obtained from such cultures were the same as in stimulated cells, but - unsurprisingly - at far lower levels. The latter is to be expected, since B cell proliferation and Ig production *in vitro*, in the absence of stimulation, is low. Due to this, for proliferation and Ig production significant reductions for the highest DON and ZEN concentration were not reached for several read-outs and conditions, but the data show exactly the same trend.

Irrespective of the TLR-agonist used, we demonstrated that DON [0.8-1.6 µM] leads to significant decreases of the proliferation of total B cell as well as specific B cell subsets, while DOM-1 left all these parameters unaffected. Similarly, several reports demonstrated a reduction in proliferation of whole PBMC cultures following ConA stimulation, which drives primarily T-cell proliferation (19, 20, 60, 61). In those studies, DON concentrations ranging from 0.33 µM to 0.94 µM started to impair cell proliferation, indicating some variability, probably depending on time of *in vitro* cultivation and methodology used for the analysis of proliferation.

With respect to ZEN, we observed that 40 µM of the mycotoxin leads to significant reductions of PBMC survival, at least when Pam3CysSKKK was used for stimulation. Furthermore, ZEN [20-40 µM] significantly reduced proliferation of total B cells as well as B-cell subsets. None of the ZEN-induced detrimental effects were observed after treatment with its metabolite HZEN. Studies have suggested that the damaging effects of ZEN on immune parameters such as cell viability and proliferation could be due to the fact that many cell types involved in the immune response have estrogenic surface receptors (62). Investigations have not only confirmed that ZEN induces apoptosis and necrosis in different immune cells, particularly in B and T cells, it has also been suggested that ZEN-induced immunosuppression is likely to be a direct result of the detrimental effects of the mycotoxin on B- and T-cell proliferation (30, 63). According to Forsell et al. (64), a study cited in the EFSA Scientific Opinion (2011) (65), proliferation of human lymphocytes stimulated with different mitogens was reduced by 50% in the presence of 3.5 µg/mL (=10.9 µM) ZEN. Furthermore, Berek et al. (63), compared the effects of ZEN and its derivatives alpha-zearalenol and ß-zeralenol with that of trichothecences on the proliferation of PBMCs, thereby showing – in accordance with our findings – that only high concentrations (>15 µM) of these toxins reduced PBMC proliferation. This was also confirmed by another investigation (66) which treated freshly isolated human PBMCs with ZEN [0.1-30 µg/mL (94 µM)] and showed proliferation inhibition and necrosis induction of B and T lymphocytes only that the highest concentration of 30 µg/mL. In comparison to ZEN, HZEN did not have any effect on the proliferation of human immune cells.

While the impacts of DON and ZEN on immune cell proliferation have been shown in our study, but also other published work, we provide new information with regard to their effects on specific B-cell subsets (CD21^+^, IgM^+^, IgG^+^). IgM is expressed as a transmembrane receptor in B1 cells and naïve B2 cells. Following recognition of cognate antigen some naïve B2 cells do not undergo class switch and can differentiate into memory B cells which still express IgM as their B-cell receptor (BCR) (67). For porcine B cells, it has been suggested that naïve B2 cells express CD21 and IgM, whereas B1 cells have an IgM^+^CD21^-^ phenotype (59). We observed that proliferation of CD21^+^ B cells was already impaired at lower concentrations of both DON [0.4 μM] and ZEN [10 μM, but only for R848 stimulation] than IgM^+^ and IgG^+^ subsets. This may indicate that CD21^+^ naïve B2 cells are more susceptible to the immunosuppressive effects of DON and ZEN than B1 cells and class-switched IgG^+^ B cells, although the underlying molecular mechanisms await elucidation. For DON, this might be due to differences in the capacity of different B-cell subsets to enter a ‘ribotoxic stress mode’ (see introduction), whereas for ZEN, differences in estrogen-receptor expression might be relevant. Nevertheless, from 0.4 μM DON and 20 μM ZEN onwards, IgM^+^ B cells also showed reduced proliferation rates and IgG^+^ B cells were affected at the highest DON and ZEN concentrations tested.

While this suggests that IgG^+^ B cells are less susceptible to DON and ZEN, their capacity for IgG secretion tested by ELISpot assays was already reduced at 20 μM of ZEN (**Figure 3B**), indicating that capacity for proliferation and antibody secretion may not respond in the same way to the actions of ZEN. Still, available ELISA tests allowed us to investigate IgM, IgA, and IgG in supernatants from the same samples (**Figure 4**) and also here, IgM and IgA production was affected at lower concentrations for DON and ZEN than IgG production.

Taken together, these findings may indicate that in particular naïve B2 cells are affected by the mycotoxins under investigation in our study. In the context of vaccination, this could imply that the induction of a primary immune response is more affected. This may result in an impaired formation of immunological memory, which in turn could lead to reduced vaccine efficacy.

In addition to providing significant information regarding the effects of DON and ZEN on cellular and humoral immune parameters, we demonstrated the DON metabolite DOM-1 and ZEN metabolite H-ZEN did not affect the above-mentioned parameters, thereby confirming the detoxifying nature of the underlying biotransformation processes. This is particularly important because even though common pre- and post-harvest mitigation strategies (68–74) as well as other technological approaches (14, 75) successfully reduce contamination levels, they fail to sufficiently clear agricultural products of mycotoxins. The use of feed additives, which enable biodegradation (38, 76, 77) or absorption (78, 79) of residual mycotoxins in the gastrointestinal tract, thereby reducing bioavailability and/or toxicity, is essential. We therefore provide valuable information, confirming the lack of negative effects of both DOM-1 and HZEN on B-cell functionality. It should be mentioned that with respect to HZEN, only a single investigation has been published, reporting a significantly reduced estrogenic effect of HZEN, compared to ZEN, in female pigs [43]. Our study is thus the first to provide information regarding the effects of HZEN on the proliferation and antibody production of immune cells.

Thus, the current study confirms that DON and ZEN, however not their derivatives DOM-1 and HZEN, compromise PBMC survival, proliferation of total B cells, as well as B cell subsets, decrease the number of IgG secreting cells and impair secretion of IgM, IgG, and IgA. We thereby not only provide additional insights regarding the negative effects of DON and especially ZEN on porcine B-cell function, we also confirm the efficacy of the detoxification process of DON to DOM-1 and ZEN to HZEN. Considering the pivotal function of B cells in the formation of protective humoral immunity, our findings emphasize the relevance of strict mycotoxin monitoring programs and the benefit of deactivators, which should contribute to improved animal health and welfare.

## Data availability statement

The raw data supporting the conclusions of this article will be made available by the authors, without undue reservation.

## Ethics statement

Ethical approval was not required for the study involving animals in accordance with the local legislation and institutional requirements because PBMCs were isolated from the blood of six 6-months-old pigs from an abattoir, which slaughters animals from different conventional finishing farms. Animals were subjected to electric high voltage anesthesia followed by exsanguination. This procedure is in accordance with the Austrian Animal Welfare Slaughter Regulation.

## Author contributions

AP: Conceptualization, Data curation, Formal analysis, Investigation, Methodology, Visualization, Writing – original draft, Writing – review & editing. AK: Data curation, Visualization, Writing – original draft, Writing – review & editing. EM: Conceptualization, Funding acquisition, Investigation, Methodology, Project administration, Resources, Supervision, Validation, Writing – review & editing. WG: Conceptualization, Funding acquisition, Investigation, Methodology, Project administration, Resources, Supervision, Validation, Writing – review & editing.
