# Supplementary material for: Effect of DON and ZEN and their metabolites DOM-1 and HZEN on B cell proliferation and antibody production
Source: Front Immunol. 2024 Feb 21;15:1338937. doi: 10.3389/fimmu.2024.1338937 (PMC10915041; doi:10.3389/fimmu.2024.1338937)
Supplement: Supplementary file 1 [file DataSheet_1.docx]

Supplementary Material

Effect of DON and ZEN and their metabolites DOM-1 and HZEN on B cell proliferation and antibody production

Alix Pierron^†^, Alexandra Kleber^†*^, Elisabeth Mayer, Wilhelm Gerner

*** Correspondence:** Alexandra Kleber; Alexandra.Kleber@dsm.com

# Supplementary Figures


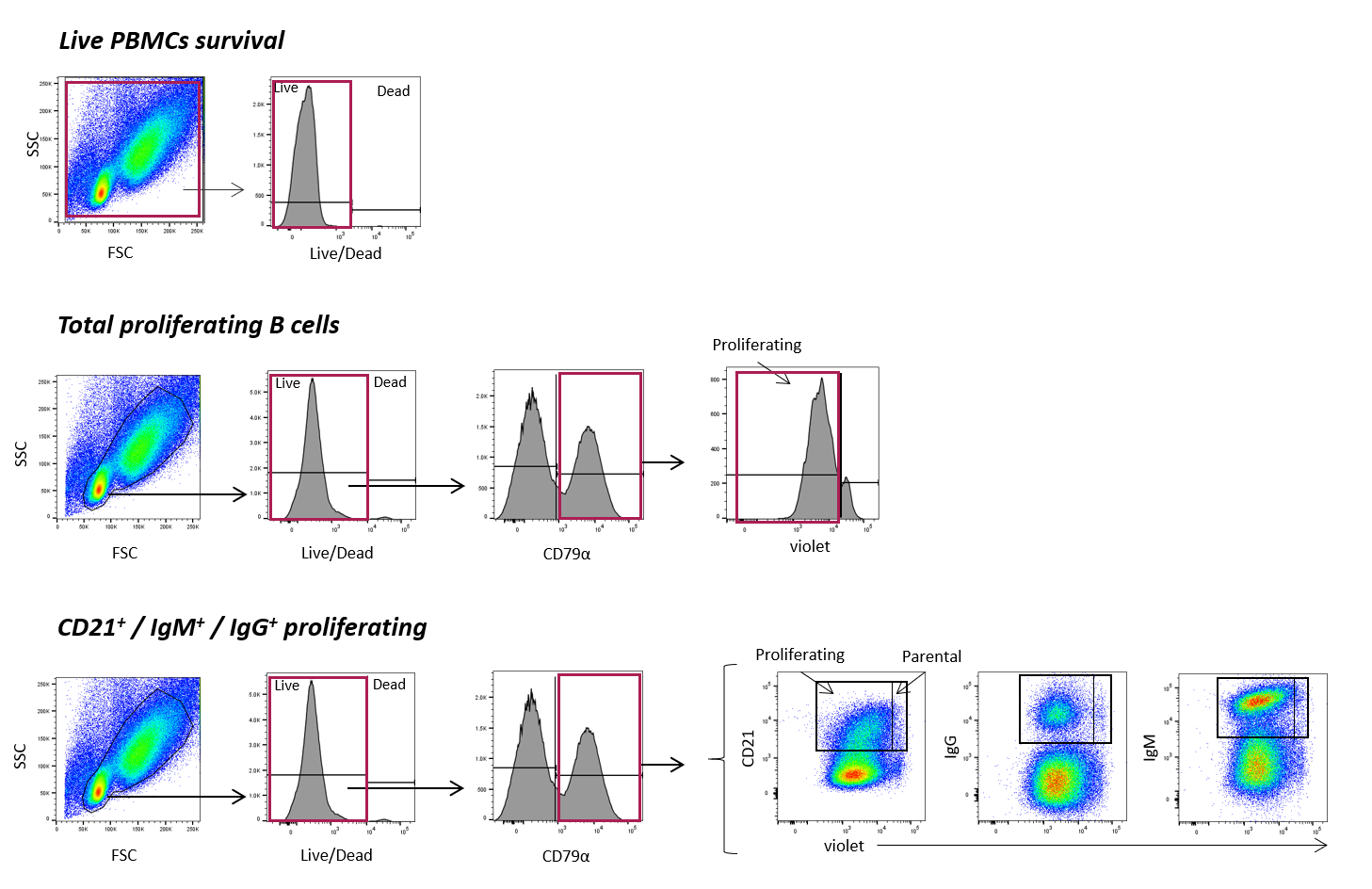


**Supplementary Figure 1.** Gating strategies for live PBMCs and proliferating B cells. Top panel: Live PBMC were identified by a large rectangular gate on FSC/SSC signal intensities to allow analysis of all cellular events. These cells were investigated for staining with the Fixable Viability dye eFluor780. Middle panel: Identification of of total proliferating B cells; lymphocytes and lymphocyte blast cells were gated based FSC/SSC properties, followed by dead cell exclusion. Total B cells were identified by expression of CD79α and proliferationg cells were identified by a decrease in violet proliferation dye within this cell subset. Bottom panel: as above but within CD79α^+^ cells proliferation of CD21^+^, IgG^+^ and IgM^+^ B cells was quantified.


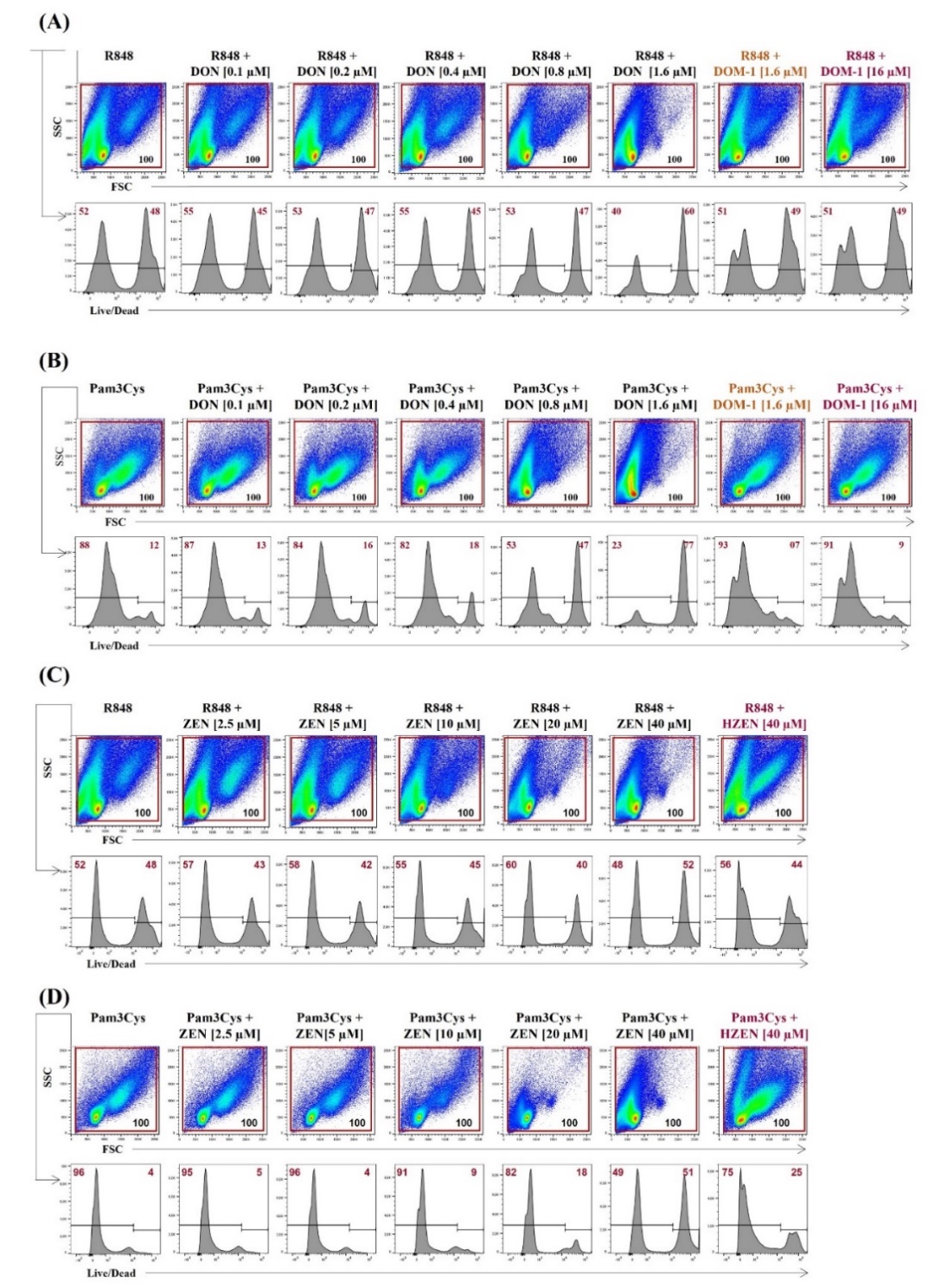


**Supplementary Figure 2.** Flow cytometry pseudocolor plots and histograms show representative raw data and gates applied to determine the survival rate. Violet proliferation dye-stained PBMCs were cultivated for four days, under R848 or Pam3Cys-SKKKK stimulation in the absence or presence of (A-B) DON [0.1-1.6 µM] or DOM-1 [1.6 and 16 µM] or (C-D) ZEN [2.5-40 µM] or HZEN [40 µM]. Cells were then harvested and labelled for live/dead discrimination and B-cell phenotyping. Representative data from one animal out of six are shown (out of three for metabolites).


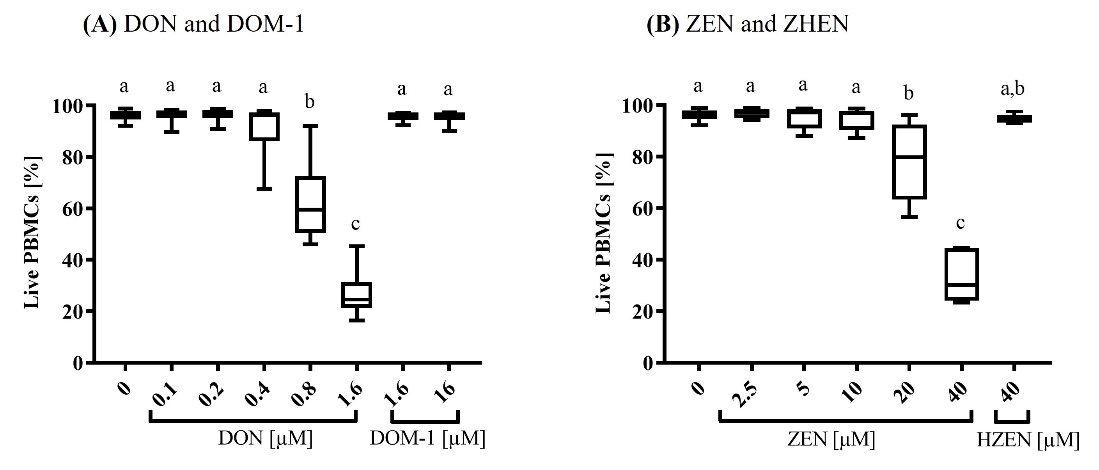


**Supplementary Figure 3**. Percentages of live but unstimulated PBMCs (i.e. no TLR-agonist) in the presence of (A) deoxynivalenol (DON) or deepoxynivalenol (DOM-1) and (B) zearalenone (ZEN) or hydrolyzed zearalenone (HZEN). PBMCs stained with a violet proliferation dye were cultured in the absence or presence of DON, DOM-1, ZEN or HZEN for a period of 4 days. Cells were then harvested and analyzed by flow cytometry. Boxplots display the percentage of live PBMCs in the presence of different mycotoxins concentrations. Different letters indicate significant differences compared to the control (One Way ANOVA, Bonferroni post-hoc test, p<0.05, n=6 for the mycotoxins and n=3 for the metabolites).


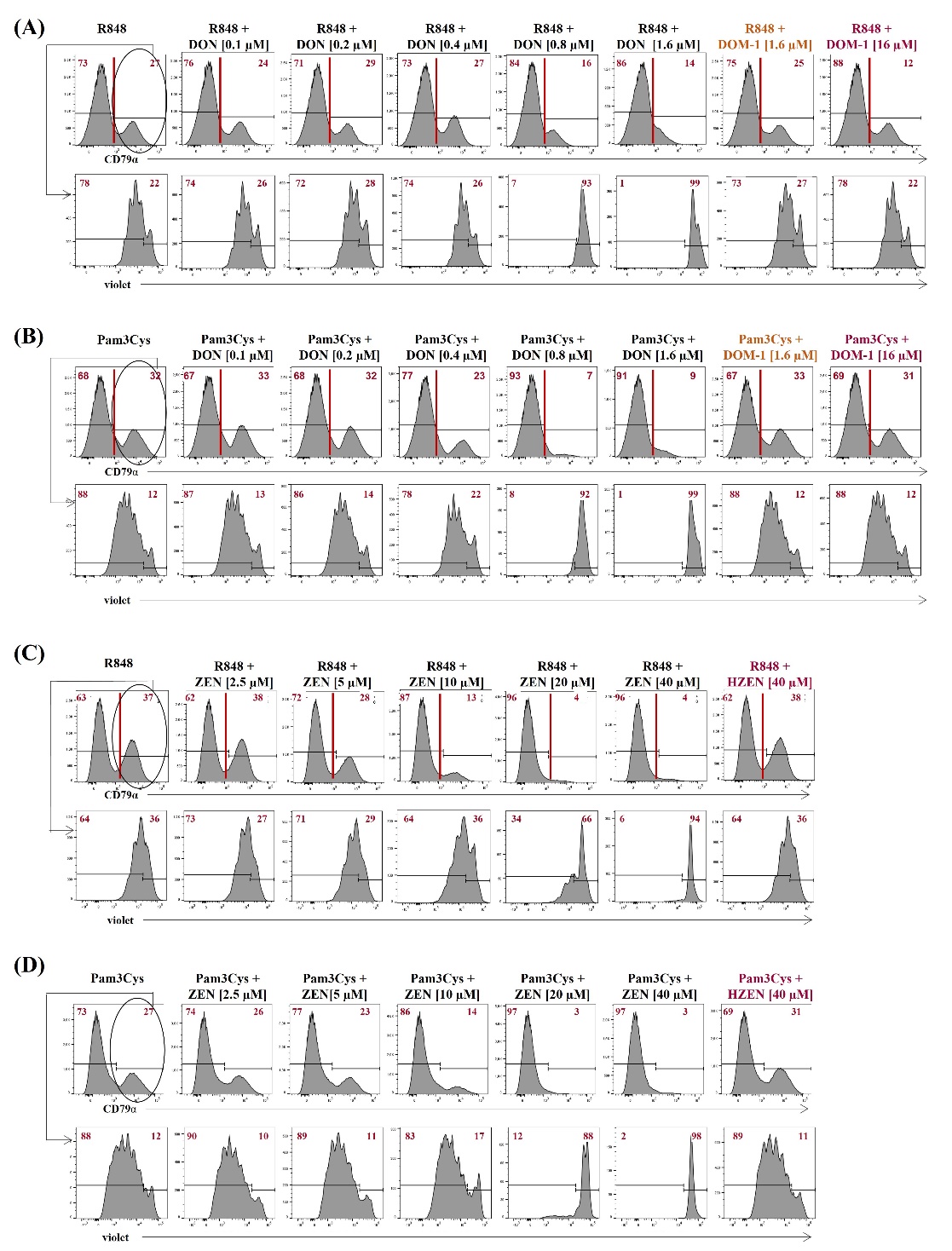


**Supplementary Figure 4.** Histograms show the fluorescence intensities of the violet proliferation dye (bottom) gated on total live CD79^+^ B-cells (top). Solid horizontal lines indicate the parental generation on the right and the proliferating generations on the left. Numbers located in the two upper corners of the histograms indicate the frequency in % of the proliferating populations in comparison to the nonproliferating cells. (A-B) Cells stimulated with R848 or Pam3CysSKKKK in the presence or absence of DON [0.1-1.6 µM] or DOM -1 [1.6 and 16 µM]. (C-D) Cells stimulated with R848 or Pam3CysSKKKK in the presence or absence of ZEN [2.5-40 µM] or HZEN [40 µM]. Representative data from one animal out of six are shown (out of three for metabolites).


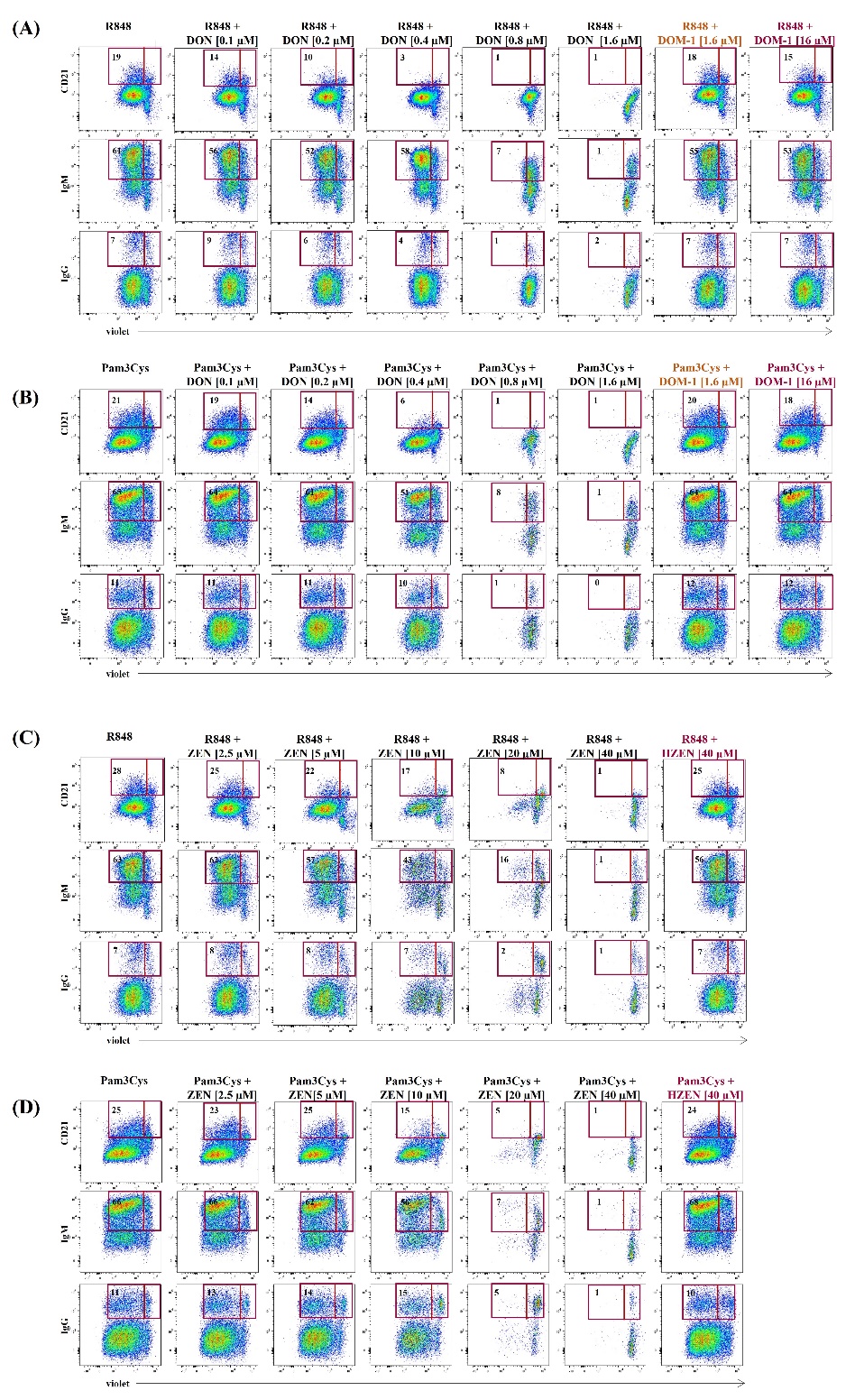


**Supplementary Figure 5.** Flow cytometry pseudocolor plots show representative raw data and gates applied to determine proliferating and non-proliferating CD21+, IgG+ or IgM+ CD79α+ B cells Violet proliferation dye-stained PBMCs were cultivated for four days under R848 or Pam3Cys-SKKKK stimulation in the absence or presence of (A-B) DON [0.1-1.6 µM] or DOM-1 [1.6 and 16 µM] or (C-D) ZEN [2.5-40 µM] or HZEN [40 µM]. Gates to the left are the proliferating cells with numbers inside indicating the percentage of proliferating cells. Representative data from one animal out of six are shown (out of three for metabolites).


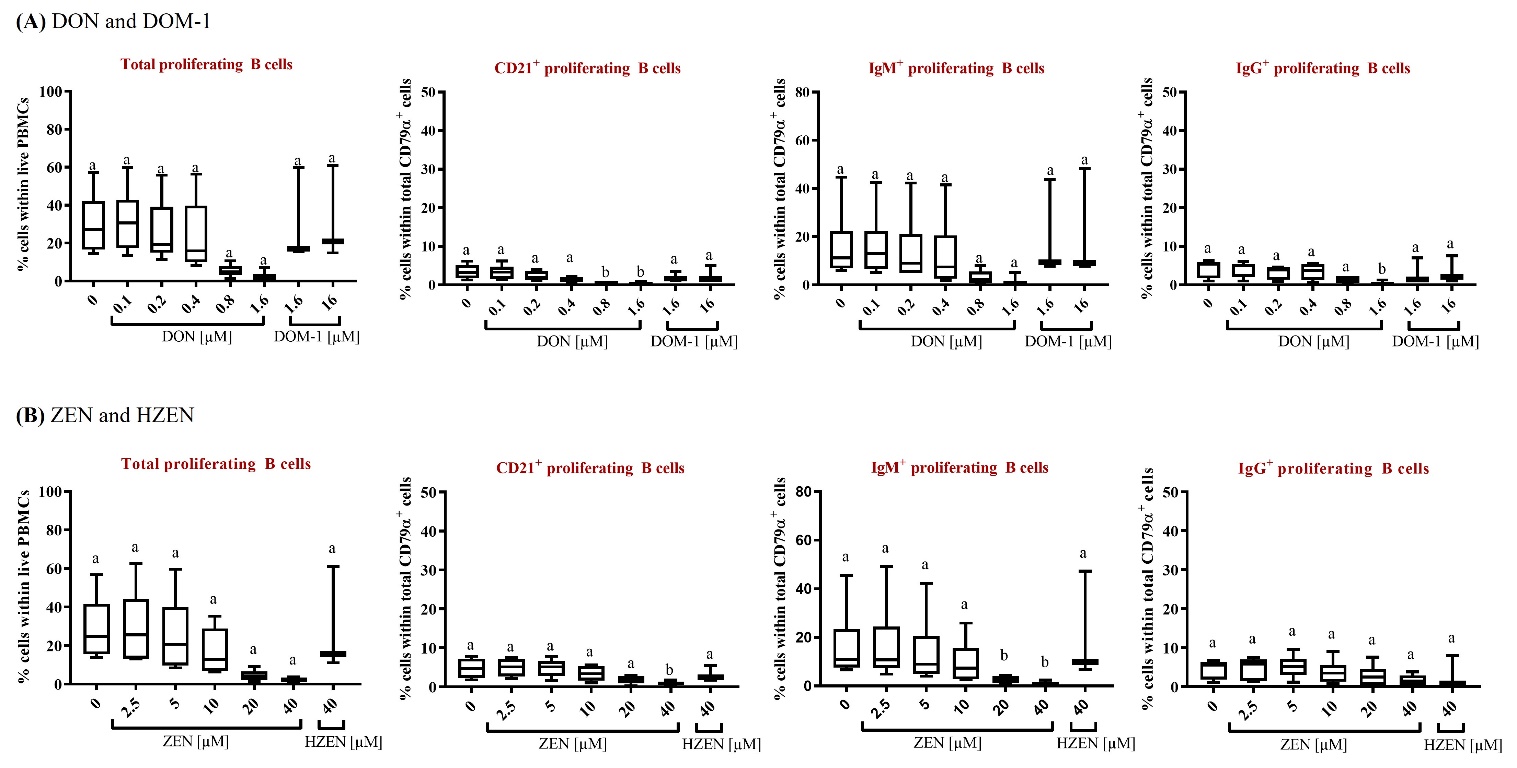


**Supplementary Figure 6.** Percentages of total proliferating B cells and major subsets (CD21^+^, IgM^+^, and IgG^+^) in the presence or absence of **(A)** DON [0.1-1.6 µM] and DOM-1 [1.6 and 16 µM] or **(B)** ZEN [2.5-40 µM] and HZEN [40 µM] without TLR-agonist stimulation. Violet proliferation dye-stained PBMCs were cultivated in the absence or presence of DON, DOM-1, ZEN, or HZEN for four days. After harvesting, cells were labeled for CD79α, CD21, IgM, and IgG. Gates were applied to identify proliferating total B cells or B-cell subsets within total B cells. Different letters indicate significant differences compared to the control (One Way ANOVA, Bonferroni post-hoc test for normally distributed data; in case normal distribution was not met, the Kruskall Wallis test was performed, p<0.05, n=6 for the mycotoxins and n=3 for the metabolites).


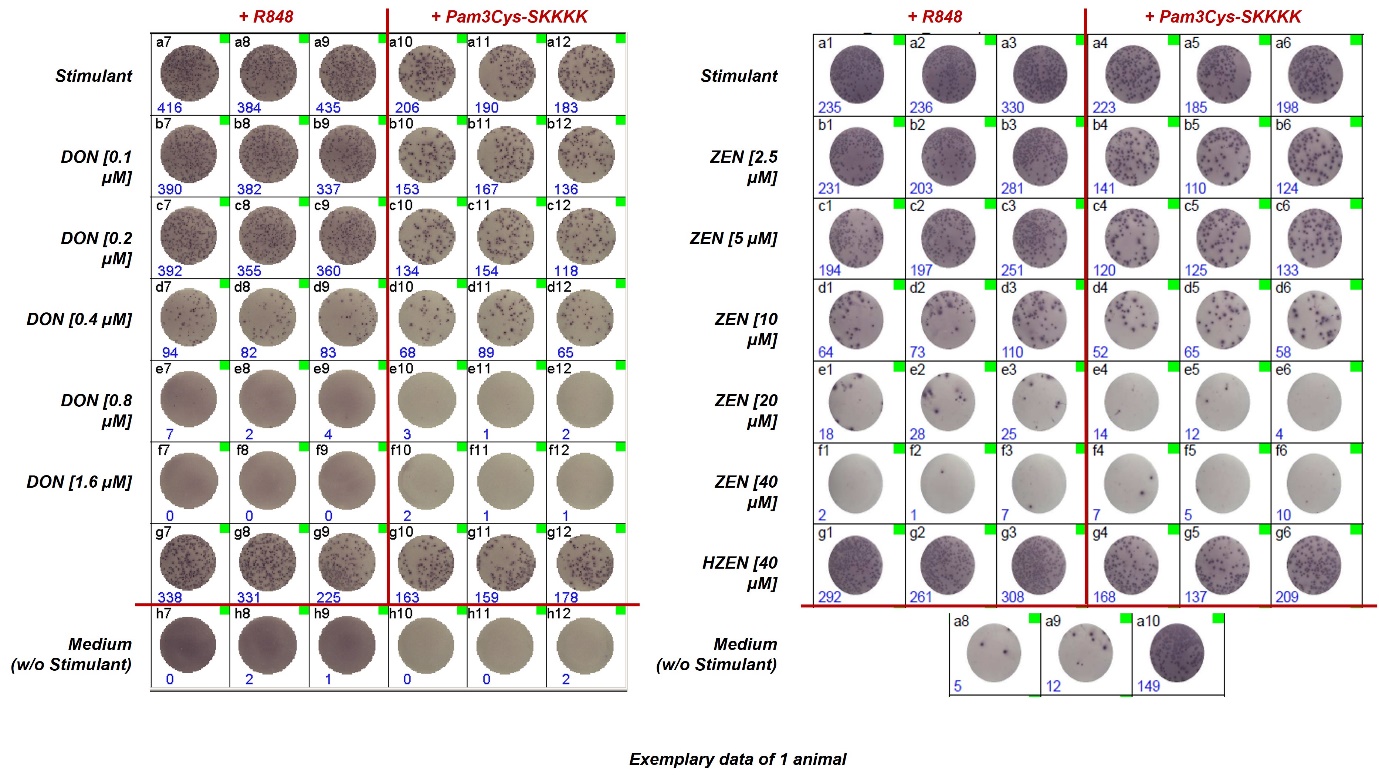


**Supplementary Figure 7.** Raw data of an IgG ELISpot assay. For each animal and each condition triplicates were analyzed. Numbers indicate the number of counted spots which represent the number of IgG secreting cells. Representative data from one animal out of six are shown.


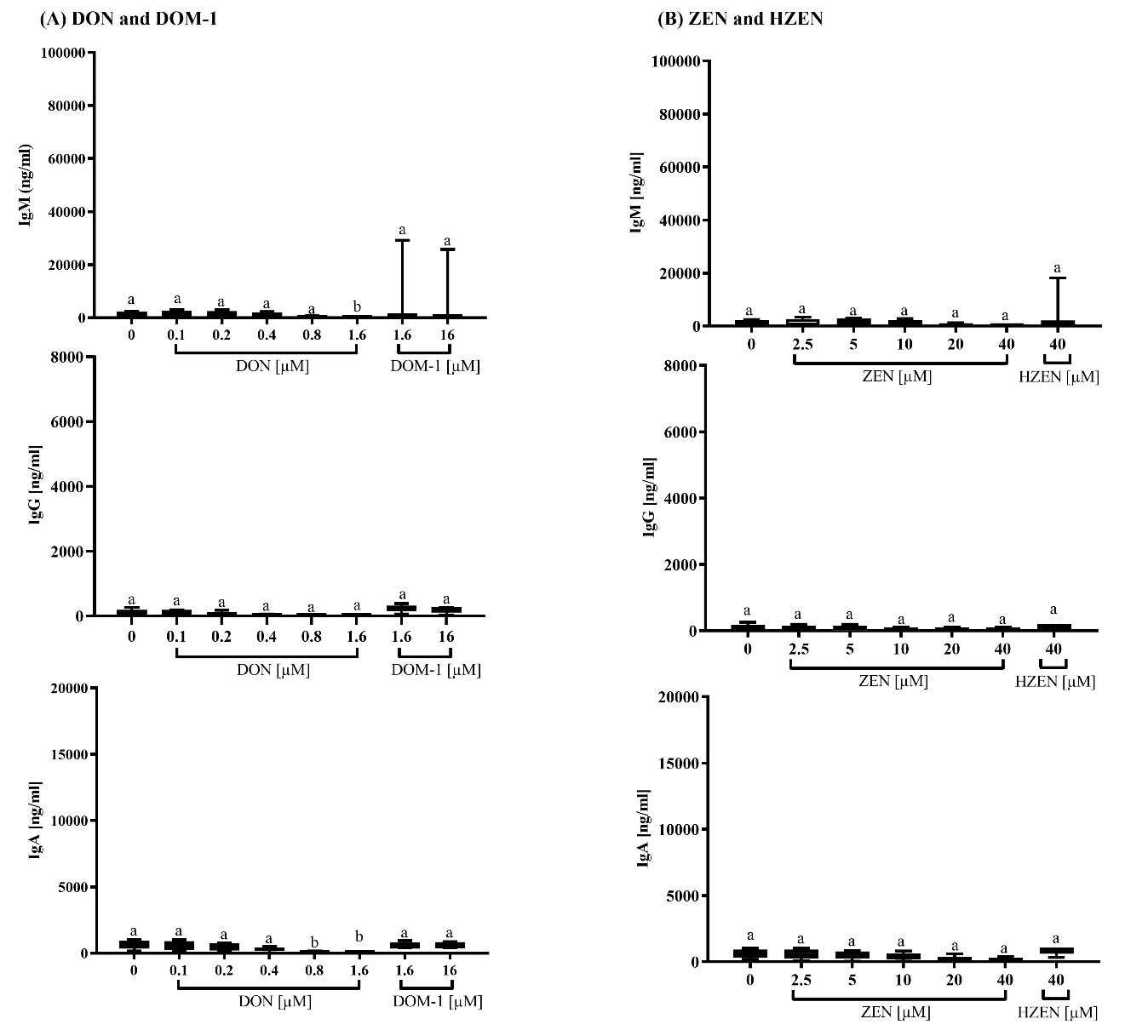


**Supplementary Figure 8.** Concentrations of IgM, IgG, and IgA antibodies in the supernatant of unstimulated PBMCs (i.e. no TLR-agonist), following treatment with **(A)** DON [0.1-1.6 µM] or DOM-1 [1.6 and 16 µM] as well as **(B)** ZEN [2.5-40 µM] or HZEN [40 µM]. PBMCs were cultivated for 4 days in the presence or absence of DON, DOM-1, ZEN, or HZEN. Antibody concentrations were determined in cell culture supernatants by ELISA. Different letters indicate significant differences to the control (One Way ANOVA, Bonferroni post-hoc test for normally distributed data; in case normal distribution was not met, the Kruskall Wallis test was performed, p<0.05, n=6 for the mycotoxins and n=3 for the metabolites).
